# The role of *Sergentomyia schwetzi *in epidemiology of visceral leishmaniasis in Ethiopia

**DOI:** 10.1186/1756-3305-7-S1-O5

**Published:** 2014-04-01

**Authors:** N Polanska, I Rohousova, P Volf

**Affiliations:** 1 Department of Parasitology, Faculty of Science, Charles University in Prague, Prague, Czech Republic

## 

Leishmaniasis is caused by a protozoan of the genus *Leishmania *and transmitted by the bites of phlebotomine sand flies. During the blood feeding, sand fly females inject saliva into the host thus affecting *Leishmania *transmission; in a naive host saliva enhances parasite virulence, in preexposed host it acts as the protective immunogenic agent by eliciting anti-saliva specific cellular and antibody immune response. Interestingly, anti-saliva antibodies in bitten hosts can be used in epidemiological studies as the marker of exposure and the risk marker of *Leishmania *transmission.

Ethiopia is endemic for visceral leishmaniasis caused by *Leishmania donovani *and transmitted mainly by *Phlebotomus orientalis*. However, the most abundant sand flies in the area belong to the genus *Sergentomyia*. *Sergentomyia *females prefer to feed on reptiles, but several studies reported mammals as the additional blood source. The main aim of this study was to determine, whether *S. schwetzi *frequently bite domestic animals and thus may play some role in the pathogen transmission.

Sera of domestic animals collected in three leishmaniasis foci were tested for anti-*S. schwetzi *IgG antibodies by ELISA using *S. schwetzi *salivary gland homogenate as an antigen. Altogether we tested 603 serum samples from five species: cattle, sheep, goats, donkeys and dogs. Sera of animals from nonendemic countries served as a negative control and the results were statistically evaluated.

Significant seropositivity for anti-*S. schwetzi *IgG was found in about one third of domestic animals tested. The highest seropositivity was found in sheep (115/181), cattle (25/108) and goats (26/144), followed by donkeys (2/24) and dogs (10/37).

Our results suggest that sand flies of the genus *Sergentomyia *frequently bite domestic animals in Ethiopia. However, further studies are needed to investigate the role of *Sergentomyia *in transmission cycle of veterinary important pathogens, including *Leishmania *sp.

The study was supported by following grant projects: Grant Agency of Charles University 675012/2012, Czech Science Foundation 13-05292S and Bill and Melinda Gates foundation.

